# Carotid body tumor imaging: MRI, ultrasound, and elastography with surgical management

**DOI:** 10.1016/j.radcr.2024.08.126

**Published:** 2024-09-21

**Authors:** Devyansh Nimodia, Shivali V. Kashikar, Pratapsingh Hanuman Parihar, Vadlamudi Nagendra, Sakshi Dudhe

**Affiliations:** Department of Radiodiagnosis, Datta Meghe Institute of Medical Sciences, Sawangi, Wardha, Maharashtra 442001, India

**Keywords:** Carotid body tumor, MRI, Ultrasound, Elastography, Postoperative

## Abstract

A carotid body tumor is an uncommon tumor that develops from the carotid body. Carotid body tumor, also called paraganglioma, is often benign in nature and mostly found in the neck. They make up 0.5% of all body tumors and resemble glomus jugulare, glomus tympanicum, and pheochromocytoma, which are paragangliomas of the body. We present a case of a 22-year-old male patient who presented to the medical outpatient department with complaints of swelling in the left carotid triangle for 1 month. The patient had hoarseness of voice with odynophagia and dysphagia. We found out the diagnosis of carotid body tumor when the patient came for ultrasound, and the diagnosis was later confirmed on magnetic resonance imaging. The gold standard treatment for carotid body tumors is surgery. The surgical categorization by Shamblin et al. marks a turning point in the evaluation of these tumors’ resectability and is still used to predict vascular morbidity, and according to it, our patient later underwent sub adventitial tumor excision.

## Introduction

An uncommon tumor that develops from the carotid body, paragangliomas make up 0.5% of all body tumors and are often benign. They make up 60%-70% of head and neck paragangliomas and resemble glomus jugulare, glomus tympanicum, and pheochromocytoma, which are paragangliomas of the body [1].

The tumor often manifests as a slow-growing, nontender, rubbery mass at the level of the hyoid, anterior to the sternocleidomastoid muscle [2]. The tumor mass may show a bruit or thrill, transmit the carotid pulse, and be movable in the lateral plane but immobile in the vertical plane. If the tumor grows around the carotid vessels and X-XII cranial nerves, dysphagia, odynophagia, and hoarseness are seen [2]. The size of the tumor also affects how it is managed since Horner's syndrome, caused by invasion or compression of the cervical sympathetic chain and syncope owing to compression of the internal carotid artery or carotid sinus, have also been reported [3]. The tumors are often well-circumscribed and may have a pseudocapusle upon gross examination. Vagus nerve palsies are the most prevalent kind of cranial nerve palsies, occurring in 30% of cases [4]. Palpitations, flushing, obstructive sleep apnea, and variable hypertension are all symptoms of catecholamine production [5].

According to research by Arya et al., the degree of circumferential contact between the paraganglioma and internal carotid artery on axial imaging may be used to predict the Shamblin group using preoperative MR imaging. The criterion to predict the Shamblin group is to find the maximum degree of circumferential contact of the carotid body tumor with the internal carotid artery on axial images. Our patient, based on this classification, was diagnosed as Shamblin type II. Morbidity to these vessels is predicted by the amount of circumferential contact with the external carotid artery and, if relevant, the common carotid artery. Finally, if carotid sacrifice is unavoidable, measuring the distal free section of the internal carotid artery is essential information to plan the viability of vascular reconstruction [6].

## Case report

A 22-year-old male patient presented to the medicine outpatient department complaining of swelling in the left lower side of the jaw for 1 month. The patient also complains of mild difficulties and pain in swallowing. He also had hoarseness of voice. The patient also complains of headaches for 1 month. The patient had no co-morbidities. The patient gave a negative history of smoking and alcohol addiction.

The patient came with chief complaints of odynophagia and dysphagia, which is seen if the tumor enlarges around the carotid vessels and X–XII cranial nerves. He also had hoarseness of voice. On inspection, the patient had swelling in the left carotid triangle. The swelling was initially small in size and was ignored by the patient, but it gradually increased, and it started causing clinical symptoms. On palpation, the swelling was firm and nontender. He also had left jugulodigastric lymphadenopathy.

**Fig. 1:**
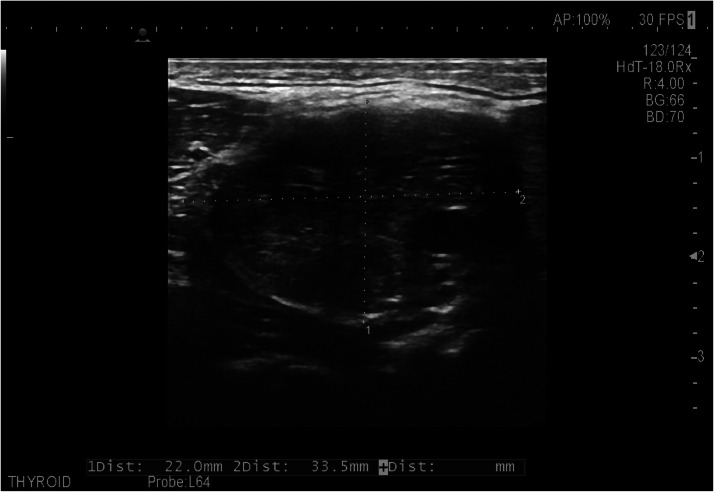
Grey scale ultrasound image showing well defined isoechoic mass in left carotid triangle measuring 33.5 × 22 mm.

**Fig. 2:**
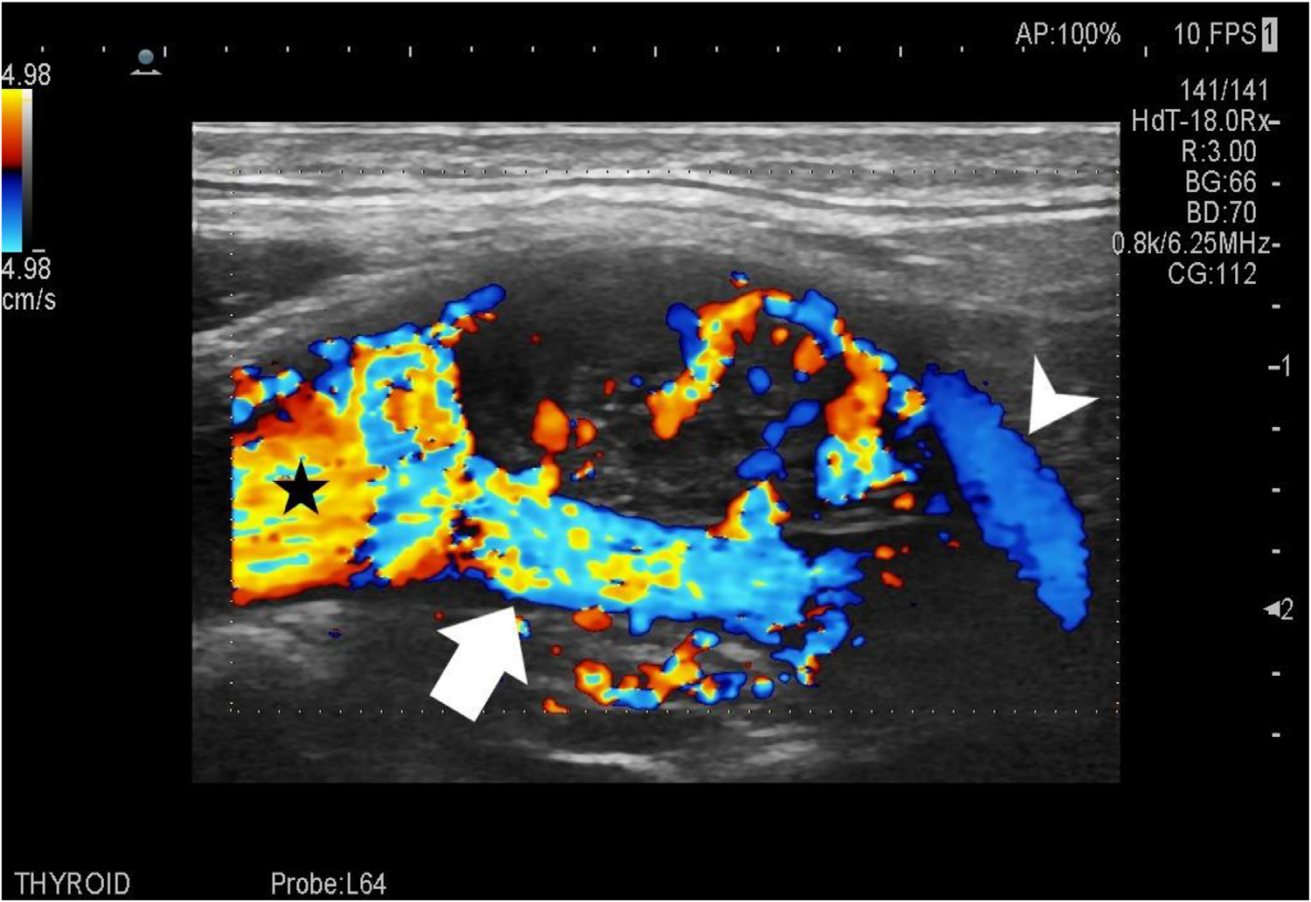
Color Doppler image of the lesion at left carotid bifurcation (asterisk showing common carotid artery) with internal vascularity. The lesion was causing splaying of ICA (arrowhead) and ECA (arrow). Legend: ICA, Internal carotid artery; ECA, External carotid artery.

On ultrasonography- A well-defined isoechoic soft tissue lesion measuring 33.5 × 22 mm is seen in the left carotid triangle (Fig. 1) at the bifurcation of the common carotid artery, causing splaying of the internal and external carotid artery. The lesion showed prominent internal vascularity on the color Doppler (Fig. 2). Strain Elastography revealed predominantly green to blue uptake, with the strain ratio being 0.07.

**Fig. 3:**
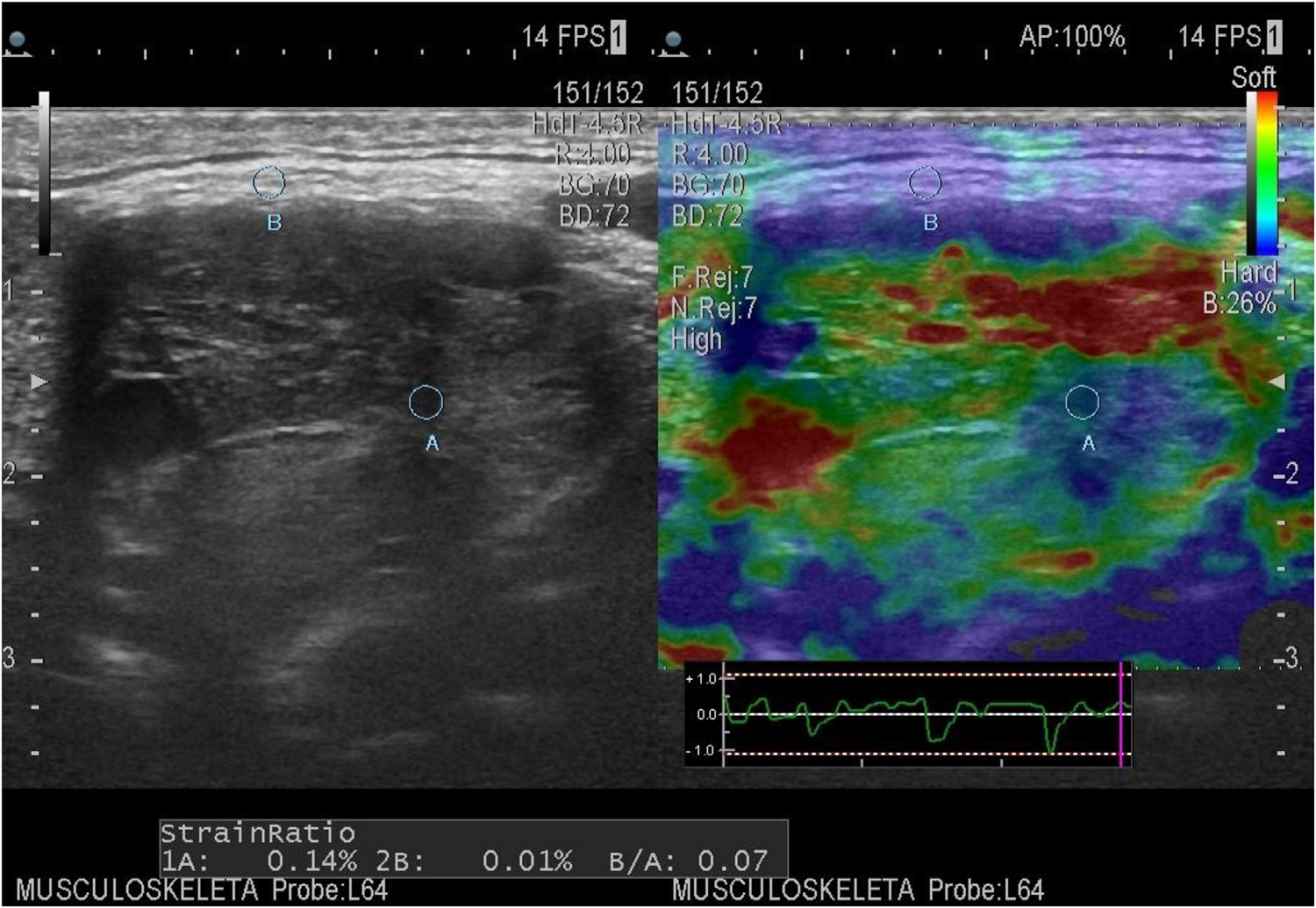
Strain Elastography of the carotid body tumor showing mosaic pattern with areas of green and blue (Tsukuba score 2) with strain ratio of 0.07 suggesting soft lesion.

Our patient belonged to elasticity score grade 2, which was of Mild stiffness and predominantly green with few red areas comprising less than 50% on US strain elastography. The mass was partially delineated from surrounding tissues. Strain Elastography of the carotid body tumor shows a mosaic pattern with areas of green and blue (Tsukuba score 2) with a strain ratio of 0.07, suggesting a soft lesion (Fig. 3).

**Fig. 4:**
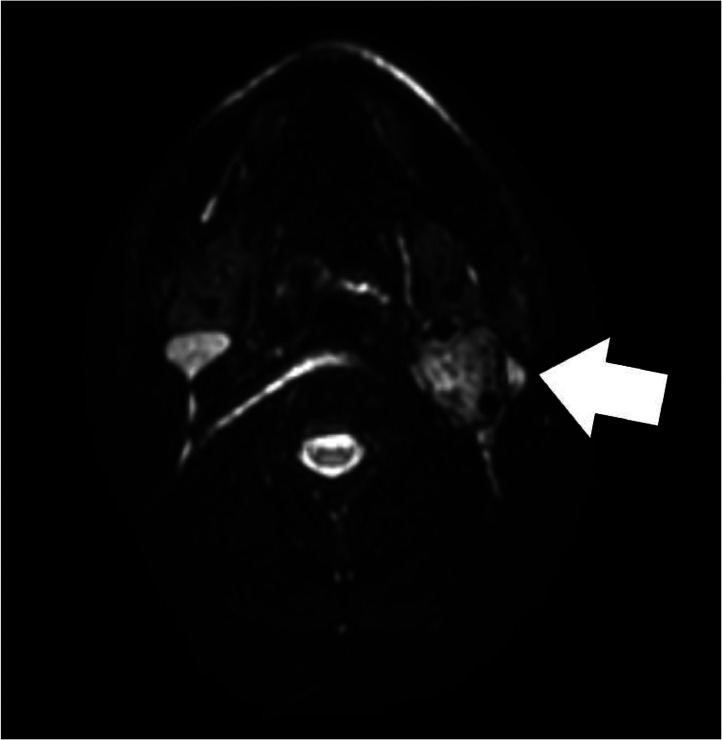
STIR DWI axial MRI neck depicting well-defined lesion in left carotid triangle showing restriction. Legend: STIR, Short tau inversion recovery; DWI, Diffusion weighted imaging; MRI, Magnetic resonance imaging.

**Fig. 5:**
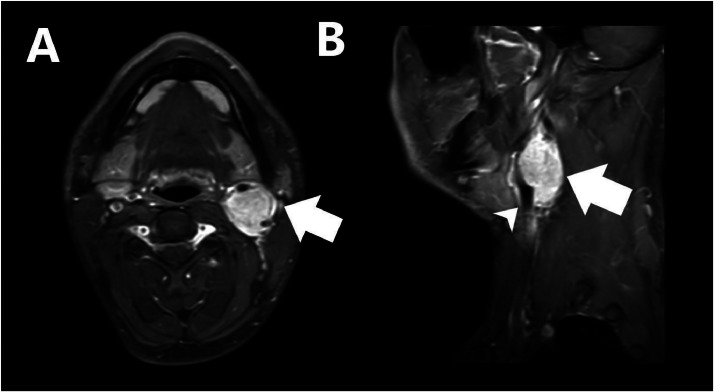
T1 Contast Axial (A) and Sagittal (B) MRI neck showing enhancing mass lesion (arrow) in left carotid triangle at the left common carotid artery bifurcation (arrowhead) causing splaying of ECA and ICA. Legend: T1 contrast, T1 weighted image contrast; MRI, Magnetic resonance imaging; ICA, Internal carotid artery; ECA, External carotid artery.

Magnetic resonance imaging (MRI) scans are excellent tools for diagnosing a carotid body tumor, as they can produce detailed images of the blood vessels as well as the tumor itself. On magnetic resonance imaging, the T1WI image showed the lesion as iso to hypointense compared to muscle. On T2WI, the lesion appeared hyperintense compared to the muscle. Also, there is intense enhancement following gadolinium. On STIR, DWI axial Magnetic resonance imaging of the neck depicted a well-defined lesion in the left carotid triangle showing restriction (Fig. 4). Contrast Axial (A) and Sagittal (B) Magnetic resonance imaging of the neck showing enhancing mass lesion (arrow) in the left carotid triangle at the left common carotid artery bifurcation (arrowhead) causing splaying of external and internal carotid artery (Fig. 5). There was splaying of the left carotid triangle at the left common carotid artery bifurcation, causing splaying of the external and internal carotid artery, which is classically described as the Lyre sign.

Based on clinical and radiological examination, a diagnosis of carotid body tumor was made. The patient was advised to undergo a surgical operation which is the primary management. Our patient fell in type 2 Shamblin on magnetic resonance imaging where the lesions partially surround the vessels and are more adherent to the adventitia, and sub-adventitial excision of the tumor was done.

**Fig. 6:**
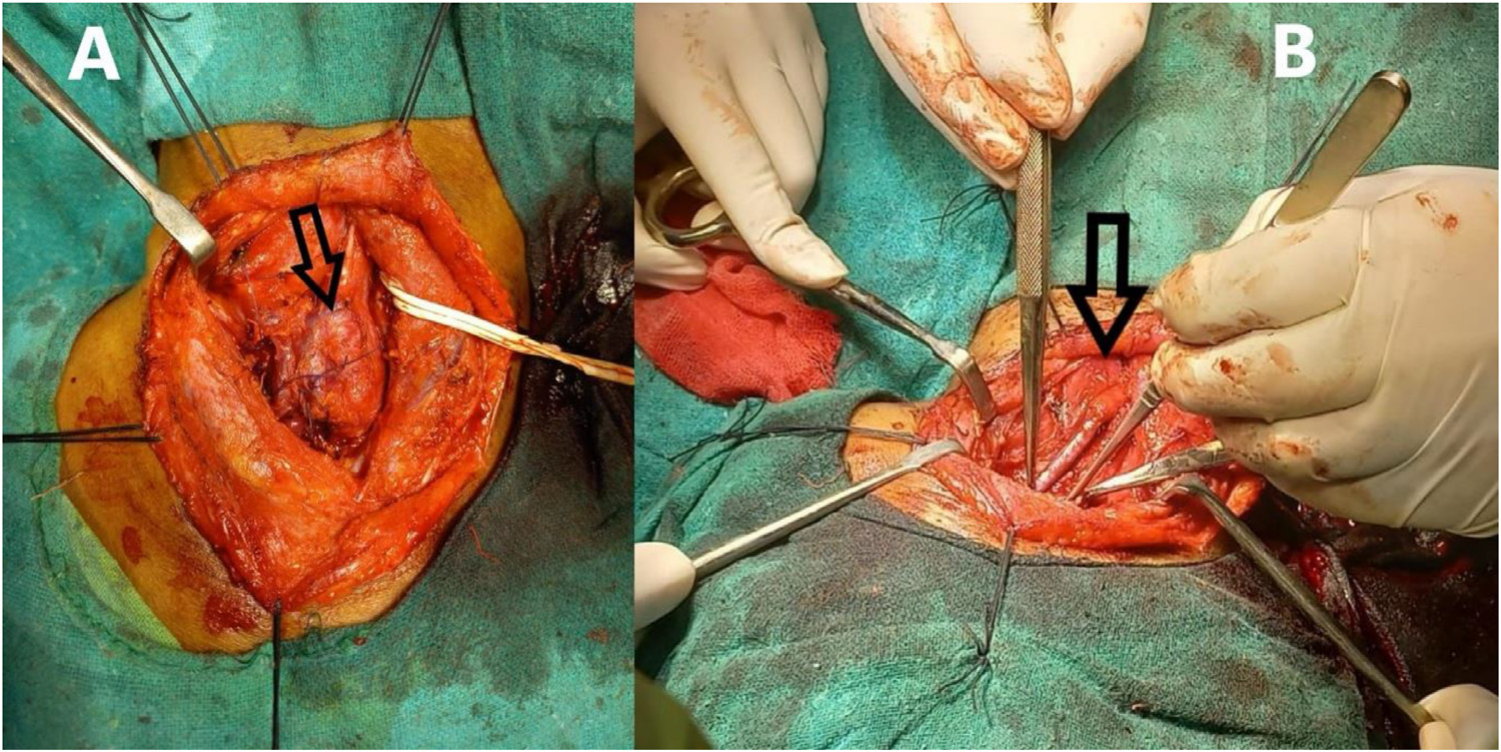
(A) Intra operative image of neck dissection showing carotid body tumor (arrow). (B) Post operative image of carotid triangle showing non visualization of carotid body tumor (arrow) with carotid defect repaired with saphenous vein graft.

After 1 week, a surgical fitness procedure of the patient was performed, and the following day surgical intervention of the tumor was done. Sub adventitial excision of the tumor was done with saphenous vein graft repair of carotid defect (Fig. 6), and the patient was advised to rest following surgery. A routine follow-up after 1 month was advised.

On discharge, the patient was advised to rest. He was also advised to come for follow-up after 1 month.

## Discussion

Neoplasms of the paraganglia within the paravertebral sympathetic and parasympathetic chains are referred to as extra-adrenal paragangliomas. Extra-adrenal paragangliomas can arise anywhere along these tracts. Some common sites of occurrence include the carotid body, retroperitoneum, mediastinum, abdomen, and chest. Some various neck and head locations include the orbit, nasopharynx, jugulotympanic membrane, vagal body, and larynx [6].

Carotid body tumor clinically presents as a slow-growing rounded neck mass. It is often situated at the level of the hyoid bone, anterior to the sternocleidomastoid, close to the mandibular angle. Due to the tumors’ position inside, the carotid sheath can only be moved side to side and not up or down.

If the tumor grows around the carotid vessels and X-XII cranial nerves, dysphagia, odynophagia, and hoarseness are seen [2]. The size of the tumor also affects how it is managed since Horner's syndrome, caused by invasion or compression of the cervical sympathetic chain and syncope owing to compression of the internal carotid artery or carotid sinus, have also been reported [3].

Glossopharyngeal, vagus, accessory, and hypoglossal cranial nerves—which travel through the carotid sheath—might be affected. Their functioning is related to the accompanying symptoms. Although less often than with adrenal paragangliomas (pheochromocytomas), these tumors may produce and release catecholamines. Vagus nerve palsies are the most prevalent cranial nerve palsies, occurring in 30% of cases. Palpitations, flushing, obstructive sleep apnea, and variable hypertension are all symptoms of catecholamine production.

Recent research points to germline mutations as the molecular foundation for developing several paragangliomas. There are 6 known genes that are considered to play a role in the growth of paraganglioma and pheochromocytomas: VHL*,* RET, SDH and NF1 and SDH subunits SDHB*,* SDHC and SDHD*.* A significant percentage of head and neck paragangliomas are caused by SDHB and SDHD mutations. Paragangliomas are well-known for having a hereditary component and being a part of genetic disorders such as tuberous sclerosis, MEN 2A, and MEN 2B, neurofibromatosis type I (von Recklinghausen disease) and Von Hippel-Lindau syndrome. Numerous family instances, frequently linked to germline abnormalities, go undiagnosed when symptoms of these more well-known syndromes are absent. When features of these more commonly known syndromes are not present, many familial cases, often associated with the above-mentioned germline mutations, go unrecognized [6].

Up to 70% of extra-adrenal paragangliomas originate from paragangliomas that arise from these parasympathetic sites, which are related to the parasympathetic nerve system in the head and neck area. The carotid body is the most frequent location. Classic radiographic characteristics can be seen in carotid body paragangliomas, which develop where the internal and external carotid arteries split. Carotid body paragangliomas present on imaging as vascular lesions, which is consistent with their nature. Internal (ICA) and external (ECA) carotid arteries are splayed out by these lesions, which encase but do not constrict the internal carotid artery and external carotid artery as they grow. The lesions exhibit an aggressive enhancement after contrast injection, which reflects their vascular character. The preferred course of therapy is surgical resection; however, because these tumors are so vascular, resection is difficult. Shamblin et al. created a surgical categorization system to better predict surgical morbidity, which is associated with the interaction of the tumor with the carotid vessels. Tumors as big as 10 cm have been recorded by some. Jugulotympanic paragangliomas are among the difficult-to-access paragangliomas that are more likely to be fragmented with unclear histologic characteristics [6].

While using USG elastography, elastograms were scored using a basic 4-point scale (ES 0-3, Elasticity grading system for cervical neck masses) comparing the lesion to surrounding subcutaneous fat and other soft tissues excluding muscle, in the lack of universally accepted scoring criteria (Table 1). This scale was developed using data from earlier thyroid US elastography research (Lyshchik et al. 2005; Itohet al. 2006; Rago et al. 2007; Asteria et al. 2008; Hong et al. 2009; Rubaltelli et al. 2009). To gain a general sense of the stiffness of lesions throughout compression-decompression cycles, which would not always be depicted on a static image due to brief fluctuations, whole cineloop segments were examined as opposed to partial cineloop segments in prior investigations [7]. Our patient belonged to ES grade 2, which was of Mild stiffness and predominantly yellow or green with few red areas comprising less than 50% on elastography. The mass was partially delineated from surrounding tissues.

**Table 1:** The elasticity grading system for cervical neck masses [7].

Elasticity score	Overall impression	Elastographic appearance of the mass
ES 0	Softer than surrounding tissues	Mass contains purple areas compared with surrounding tissues that are displayed predominantly green, yellow or red. Lesion can be clearly delineated from surrounding tissues.
ES 1	Soft as surrounding tissues	Predominantly green or yellow and is indistinguishable from surrounding tissues.
ES 2	Mild stiffness	Predominantly yellow or green with few red areas comprising less than 50%. Mass is partially delineated from surrounding tissues.
ES 3	Stiff	Predominantly red (over 50%) and is distinguishable from surrounding tissues.

Histologically, paragangliomas, in general and carotid body tumors in particular show a distinctive development pattern known as a "zellballen" growth pattern [6].

Paragangliomas of the head and neck and carotid body tumors are often painless, slow-growing tumors that are frequently present for years before the patient seeks medical care. They might develop to be very big, and infiltrative growth and local recurrence could be fatal. Up to 30% of clinical diagnoses are incorrect, which might result in unnecessary attempts at biopsy or exploratory surgery [8].

**Fig. 7:**
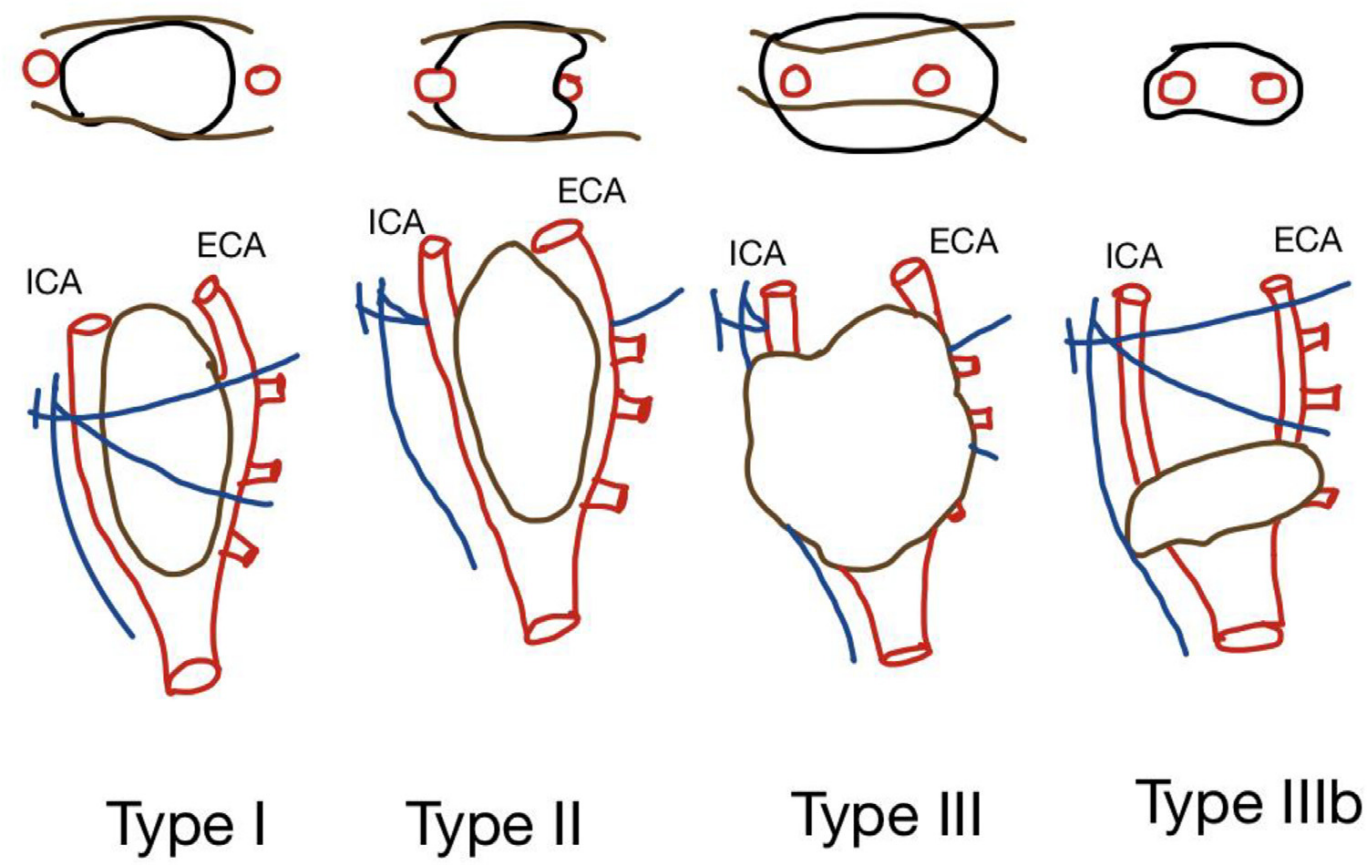
Classification of shamblin group of CBT from I to III. The surgical grouping is chiefly based on the relationship of the tumor to the carotid vessels, ICA and ECA. The oblique lines shown represent the X and XII nerves, which are intimately related to the tumors and must be carefully dissected along with the vessels. Legend: The criterion to predict the Shamblin group is to find the maximum degree of circumferential contact of the carotid body tumor with the internal carotid artery on axial images. On preoperative Magnetic resonance imaging of the tumor- The internal carotid artery with a maximum degree of circumferential contact of less than or equal to 180°, more than 180 and less than 270°, and higher than or equal to 270° would indicate Shamblin I, Shamblin II, and Shamblin III, respectively. The surgeon must be especially informed if the tumors are tiny but exhibit circumferential involvement of the internal carotid artery (stage IIIb of the proposed revision of the Shamblin classification), since the modest size may be mistaken for increased ease of removal.

According to research by Arya et al., the degree of circumferential contact between the paraganglioma and internal carotid artery on axial imaging may be used to predict the Shamblin group using preoperative magnetic resonance imaging [9]. The criterion to predict the Shamblin group is to find the maximum degree of circumferential contact of the carotid body tumor with the internal carotid artery on axial images. On preoperative magnetic resonance imaging of the tumor- The internal carotid artery with a maximum degree of circumferential contact of less than or equal to 180°, more than 180 and less than 270°, and higher than or equal to 270° would indicate Shamblin I, Shamblin II, and Shamblin III, respectively (Fig. 7). However, because greater volumes are known to be connected to time-consuming surgery and neurologic damage, the size (or volume) is a separate consideration that must be included. The surgeon must be especially informed if the tumors are tiny but exhibit circumferential involvement of the internal carotid artery (stage IIIb of the proposed revision of the Shamblin classification), since the modest size may be mistaken for increased ease of removal. Our patient fell in the type 2 shamblin group, where the lesions partially surround the vessels and are more adherent to the adventitia [9].

Because carotid artery aneurysms and elongation imitate the tumor in this region, fine needle aspiration with broad bore needle or biopsies of these tumors are risky. By using the right method, a small needle caliber, and avoiding unnecessary pricks, the increased risk of FNA-related hemorrhages can be reduced [4]. Surgical excision is the preferred method of care for paragangliomas of the carotid body. Our patient who was of shamblin grade 2, sub adventitial tumor excision of the tumor was done. Due to these tumors’ high vascularity, preoperative adrenergic inhibition should be taken into consideration, and preoperative embolization is wise. The overall outlook is quite favorable with full surgical resection. However, as metastasis and recurrence might happen years down the road, ongoing follow-up is required. Malignant paragangliomas are thought to have a 10-year survival rate of fewer than 50%. Since chemotherapy and radiation do not appear to be much more beneficial, surgery is still the preferred treatment for these malignant tumors [6].

Only CBTs that are advanced and incurable, as well as those with numerous tumors, are candidates for radiation therapy as a kind of treatment.

Our diagnosis was carotid body tumor and the general imaging differential considerations include cervical vagal schwannomas, glomus vagale, carotid bulb ectasia and vagal neurofibroma. Cervical vagal schwannoma which typically manifests as a slowly expanding, asymptomatic mass that is well-encapsulated and circumscribed. It grows 2.5 to 3 mm annually, is always closely attached to its nerve of origin and displaces the internal jugular vein laterally and the carotid artery medially. The most frequent symptom of vocal cord paralysis is hoarseness, whereas the pathognomonic indication of a vagal schwannoma is a paroxysmal cough that occurs when the mass is palpated [10] because of vagal activation. Rare reports of malignancy are often linked to neurofibromatosis type 1. Since the very first experiences, surgical excision has been the therapy of choice. Given that the tumor arises directly from the nerve fibres, vagal nerve damage is still an unresolved issue. Another differential for carotid body tumors is the glomus vagale tumor, which has the same pathology but is located more rostrally. Paragangliomas called glomus vagale tumors can develop along the vagus nerve's course (CN X). They are a subgroup of nonchromaffin paraganglion cell-derived extra-adrenal neuroendocrine tumors. usually appears as an asymptomatic tumor behind the carotid artery. Vocal cord paralysis is a condition that occurs rather frequently (47%). On MRI, the T1 sequence usually shows a low signal, the T2 signal shows a high signal with multiple flow voids, which may give a salt and pepper appearance, and on T1+contarast, there is intense enhancement. Some other differentials include carotid bulb ectasia and vagal neurofibroma.

## Conclusion

In this case, report, we have discussed carotid body tumors using clinical and diagnostic imaging, Shamblin classification using preoperative magnetic resonance imaging and ultrasound elastography, which gives us an idea about diagnosis and treatment plan. Our patient fell in the type 2 shamblin group, where the lesions partially surround the vessels and are more adherent to the adventitia.

Carotid body tumor, also called paraganglioma, is an uncommon tumor which is often benign in nature and most commonly found in the neck. The patient usually presents with pain and swelling in the neck. It is often associated with difficulty in swallowing with odynophagia and hoarseness of voice. The gold standard treatment for carotid body tumors is surgery. However, it can be difficult since the tumor is a highly vascular mass that frequently adheres tightly to the carotid bifurcation. A successful surgical outcome depends on careful patient selection and meticulous preoperative preparation.

## Ethics approval and consent to participate

Written consent taken.

## Availability of data and material

N/A.

## Author contributions

VN was involved in providing clinical details of the patient. SVK discussion on the pathology. PHP accumulated the results of the patient's radiological investigations. NR was involved in collecting images and formatting data. All authors have read and approved the manuscript.

## Patient consent

Informed and written consent was obtained from the patient.
